# Clinical Characteristics and Prognostic Risk Factors in Breast Cancer With Liver Metastasis

**DOI:** 10.1155/tbj/5088737

**Published:** 2026-06-20

**Authors:** Dandan Yu, Chengjun Zhu, Sujin Yang, Dandan Wang, Xiaoxiang Guan

**Affiliations:** ^1^ Department of Radiation Oncology, The First Affiliated Hospital of Nanjing Medical University, Nanjing, 210029, China, njmu.edu.cn; ^2^ Department of Oncology, The First Affiliated Hospital of Nanjing Medical University, Nanjing, 210029, China, njmu.edu.cn; ^3^ Department of Breast Surgery, The First Affiliated Hospital of Nanjing Medical University, Nanjing, 210029, China, njmu.edu.cn; ^4^ Jiangsu Key Lab of Cancer Biomarkers, Prevention and Treatment, Collaborative Innovation Center for Personalized Cancer Medicine, Nanjing Medical University, Nanjing, 210029, China, njmu.edu.cn

**Keywords:** breast cancer, liver metastasis, molecular subtype, receptor conversion

## Abstract

**Background:**

Liver metastasis is a key adverse prognostic factor in breast cancer patients. This research was aimed to assess the development, risk factors, and prognostic determinants of breast cancer liver metastasis (BCLM).

**Methods:**

We retrospectively analyzed the data of breast cancer from the Surveillance, Epidemiology, and End Results (SEER) (*N* = 560,908) and Jiangsu Province Hospital (JSPH) database (*N* = 294). The risk factors for BCLM were identified via multivariate logistic regression, and overall survival (OS) was assessed with Kaplan–Meier (KM) survival curves and Cox regression models.

**Results:**

In the SEER cohort, liver metastasis attacked 1.3% of patients, and the highest incidence was found in the HR−/HER2+ subtype (4.4%). The risk factors for BCLM include young age, high pathological grade, concurrent bone, lung or brain metastasis, and HER2‐positive or triple‐negative subtype. The median OS of BCLM patients was short (SEER: 22 months; JSPH: 33.5 months). OS was shorter in patients with concomitant metastasis to other organs or with HER2‐negative subtype. Hepatic resection remarkably prolonged survival (SEER: 90 vs. 35 months; JSPH: not reached vs. 31.3 months). In the JSPH cohort, molecular subtype changed in 27.5% of patients during metastasis.

**Conclusions:**

The occurrence of BCLM is affected by age, tumor grade, other organ involvement, and molecular subtype. Survival was improved in BCLM patients with liver‐only metastasis, HER2‐positive subtype, or those who underwent hepatic resection. The number and molecular characteristics of liver metastasis are important prognostic predictors, and receptor conversion highlights the need for reassessment of therapeutic strategies during metastatic progression.

## 1. Introduction

Breast cancer is the most frequently diagnosed cancer and the dominant cause of cancer‐induced deaths in women globally [1]. Recent advances in targeted and endocrine therapies have extended the survival of HER2^+^ patients and those with ER^−^/HER2^−^ breast cancer [2, 3]. However, distant metastasis is still the primary risk of breast cancer–related death. The median overall survival (mOS) of metastatic breast cancer (MBC) is about 3 years, while the 5‐year survival rate is only 25% [4]. The main metastatic organs of breast cancer are bone, lung, liver, and brain. About 30% of breast cancer patients develop liver metastasis, which makes liver the third most common metastatic site after bone and lung [5]. Liver metastasis is a key factor impacting the long‐term survival of breast cancer patients and can lead to treatment resistance and consequently a higher mortality rate. The mOS of patients with breast cancer liver metastasis (BCLM) is 14.3 months [6].

The development and prognosis of BCLM are influenced by multiple factors, among which molecular subtypes play a predominant role. The molecular subtypes of the heterogeneous breast cancer classified by hormone receptor (HR) and HER2 status include HR+/HER2‐, HR+/HER2+, HR‐/HER2+, and triple‐negative breast cancer (TNBC) [7]. HR+/HER2‐ tumors are more prone to bone metastasis, whereas HER2+ tumors and TNBC are more susceptible to visceral involvement, including liver and brain metastases [8–10]. Large‐scale population studies confirm that HER2+ and TNBC patients carry significantly increased risks of liver metastasis compared to HR+/HER2‐ patients [9, 10]. A retrospective study involving 1840 breast cancer patients shows that HER2 status, tumor size, and lymph node metastasis are linked to the development of liver metastasis [11]. A study of 1228 patients with MBC indicates that HER2 status in de novo MBC, HER2 status, and invasive ductal carcinoma histology in recurrent MBC are risk factors for BCLM [12]. However, the influence of molecular subtypes on the prognosis of BCLM patients is still under debate. In a real‐world cohort, HR+/HER2‐ breast cancer patients exhibited the longest mOS following liver metastasis (58.0 months), while TNBC patients showed the shortest (26.0 months) [13]. As reported, mOS was the longest in HR+/HER2+ BCLM patients but the shortest in those triple‐negative patients [14]. In a retrospective cohort of 104 BCLM patients, molecular subtype had no significant impact on survival after liver metastasis [15].

The studies above mostly rely on single‐center retrospective data. Although these studies provide valuable insights, research focusing on the clinical characteristics of BCLM patients or prognostic factors remains limited. The association of molecular subtypes with prognosis seemingly varies among patient populations. Here, we identified the risk factors for BCLM using data from the SEER database, and together with the Jiangsu Province Hospital (JSPH) cohort, probed into the prognostic determinants of BCLM. We also examined the conversion of molecular subtypes between primary breast tumors and liver metastatic lesions and assessed their connections with patient survival, aiming to further understand the prognostic importance of molecular subtypes.

## 2. Materials and Methods

### 2.1. Patients

Data were acquired from the SEER Program of the National Cancer Institute (https://seer.cancer.gov/) on SEER∗Stat 9.0.41. We extracted data from SEER∗Stat: Incidence—SEER Research Data, 17 Registries, Nov. 2024 Sub (2000–2022), released in April 2025 and based on November 2024 submission. Totally 823,935 breast cancer cases were initially identified. Inclusion criteria were (a) age ≥ 18 years; (b) histologically confirmed breast cancer. Exclusion criteria were (a) diagnosis of carcinoma in situ; (b) presence of two or more primary malignancies; (c) unknown follow‐up duration or survival time of 0 month; (d) diagnosis based solely on autopsy or death certificate; (e) unknown metastatic status; (f) missing survival data; (g) male patients; (h) incomplete or missing clinicopathological information. Totally 560,908 eligible patients were included, of whom 7247 patients had known liver metastasis status.

We collected data on BCLM patients at JSPH between 2015 and 2025. These patients were pathologically confirmed based on liver biopsy specimens. Totally 294 patients were enrolled in the subsequent analysis. Patient medical records were reviewed to obtain the age at the time of initial diagnosis and liver biopsy, ER, PR, and HER2 status (assessed separately for primary and liver specimens), type of surgery at diagnosis, sites of metastatic involvement, prior treatment regimens, postliver metastasis OS, and date of death or most recent follow‐up. The study protocol was approved by the Ethics Committee of JSPH (2025‐SR‐918).

### 2.2. Study Design

Totally 7247 BCLM patients identified from 560,908 primary breast cancer cases in the SEER database were included. Using these data, we examined both the risk factors linked to the development of liver metastasis and the prognostic factors influencing the survival of BCLM patients. OS was determined from the date of diagnosis to death.

We also involved 294 BCLM patients from the JSPH database. Prognostic factors impacting the survival of these patients were tested. The impacts of hepatic resection and liver metastasis molecular subtype on prognosis were further tested. Receptor status conversion between primary breast tumors and liver metastases and its association with survival were also examined. OS was calculated from the date of liver metastasis diagnosis to death from any cause.

### 2.3. Statistical Analysis

Baseline information of the study patients was summarized via descriptive analyses. Factors related to liver metastasis were identified via multivariate logistic regression, and odds ratios (ORs) and 95% confidence intervals (CIs) were reported. OS‐related prognostic factors were tested using uni‐ and multivariate Cox proportional hazard regressions, and relevant hazard ratios (HRs) with 95% CIs were determined. Survival distributions were tested with the Kaplan–Meier (KM) approach, and between‐group differences were examined via log‐rank test. All statistical analyses were completed in R 4.4.2, and two‐sided *p* < 0.05 was considered significant. Sankey diagrams were plotted on OriginPro 2025.

## 3. Results

### 3.1. Baseline Information

**FIGURE 1:**
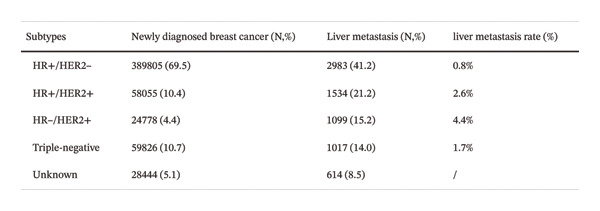
Incidence of liver metastasis in different molecular subtypes of breast cancer.

In the SEER database, totally 560,908 patients with invasive breast cancer between 2010 and 2022 were included. Among them, about 3.5% (*N* = 19,602), 1.5% (8659), 1.3% (7247), and 0.4% (2134) suffered bone, lung, liver, and brain metastases at initial diagnosis, respectively. HR+/HER2‐, HR+/HER2+, HR‐/HER2+, and TNBC accounted for 69.5% (*N* = 389,805), 10.4% (58,055), 4.4% (24,778), and 10.7% (59,826), respectively, with liver metastasis rates of 0.8% (2983), 2.6% (1534), 4.4% (1099), and 1.7% (1017), respectively (Table 1 and Figure 1). These findings indicate that among newly found breast cancers, the incidence of liver metastasis (1.3%) ranks third after bone and lung metastases. The rate of liver metastasis (4.4%) is the highest in the HR−/HER2+ subtype.

**TABLE 1:** Baseline characteristics of breast cancer with liver metastases at diagnosis in the SEER database.

Variable	Patients, no. %	
	Total (*n* = 560,908)	With liver metastases (*n* = 7247)
Age, y (%)		
18–40	39,937 (7.1)	956 (13.2)
41–60	238,146 (42.5)	3236 (44.7)
61–80	242,304 (43.2)	2575 (35.5)
> 80	40,521 (7.2)	480 (6.6)
Race (%)		
White	359,181 (64.0)	4266 (58.9)
Black	60,994 (10.9)	1285 (17.7)
Hispanic	76,757 (13.7)	976 (13.5)
Asian or Pacific Islander	57,004 (10.2)	656 (9.1)
American Indian/Alaska Native	3667 (0.7)	44 (0.6)
Unknown	3305 (0.6)	20 (0.3)
Marital status (%)		
Unmarried	222,641 (39.7)	3662 (50.5)
Married	312,193 (55.7)	3251 (44.9)
Unknown	26,074 (4.6)	334 (4.6)
Histology (%)		
Infiltrating duct carcinoma	435,002 (77.6)	5469 (75.5)
Lobular carcinoma	51,875 (9.2)	445 (6.1)
Infiltrating duct and lobular carcinoma	25,795 (4.6)	220 (3.0)
Other types	48,236 (8.6)	1113 (15.4)
Pathological grade (%)		
I	112,964 (20.1)	153 (2.1)
II	213,118 (38.0)	1286 (17.7)
III/IV	134,491 (24.0)	2062 (28.5)
Unknown	100,335 (17.9)	3746 (51.7)
Surgery of primary site (%)		
Yes	510,626 (91.0)	1378 (19.0)
No	49,275 (8.8)	5816 (80.3)
Unknown	1007 (0.2)	53 (0.7)
Radiotherapy (%)		
Yes	329,897 (58.8)	2096 (28.9)
No/unknown	231,011 (41.2)	5151 (71.1)
Chemotherapy (%)		
Yes	230,261 (41.1)	5311 (73.3)
No/unknown	330,647 (58.9)	1936 (26.7)
Bone metastasis (%)		
Yes	19,602 (3.5)	4461 (61.6)
No	541,306 (96.5)	2786 (38.4)
Brain metastasis (%)		
Yes	2134 (0.4)	704 (9.7)
No	558,774 (99.6)	6543 (90.3)
Lung metastasis (%)		
Yes	8659 (1.5)	2427 (33.5)
No	552,249 (98.5)	4820 (66.5)
Subtype (%)		
HR+/HER2‐	389,805 (69.5)	2983 (41.2)
HR+/HER2+	58,055 (10.4)	1534 (21.2)
HR‐/HER2+	24,778 (4.4)	1099 (15.2)
Triple‐negative	59,826 (10.7)	1017 (14.0)
Unknown	28,444 (5.1)	614 (8.5)

In the JSPH database, a total of 294 breast cancer patients with pathological confirmation based on liver lesion biopsy were included. In the cohort, 89.1% had recurrent MBC (*N* = 262), and the majority (93.9%) had undergone primary tumor resection. About 49.0% (*N* = 144), 28.9% (85), and 4.1% (12) had concurrent bone, lung, and brain metastases, respectively. We analyzed the molecular subtypes of both primary and liver metastatic lesions and found changes in receptor expression. Notably, the proportion of TNBC subtype markedly increased in liver metastasis (Table 2). During follow‐up, OS data were available for 167 patients.

**TABLE 2:** Baseline characteristics of breast cancer patients with liver metastases in the JSPH database.

Variable	Patients, no. %
	Patients (*n* = 294)
Age at diagnosis	
18–40	72 (24.5)
41–60	189 (64.3)
61–80	33 (11.2)
De novo metastatic diseases	
No	262 (89.1)
Yes	32 (10.9)
Surgery of primary site	
No	17 (5.8)
Yes	276 (93.9)
Unknown	1 (0.3)
Surgery of liver metastasis site	
No	259 (88.1)
Yes	35 (11.9)
Prior chemotherapy	
No	2 (0.7)
Yes	279 (94.9)
Unknown	13 (4.4)
Prior radiotherapy	
No	123 (41.8)
Yes	154 (52.4)
Unknown	17 (5.8)
Bone metastasis	
No	150 (51.0)
Yes	144 (49.0)
Brain metastasis	
No	282 (95.9)
Yes	12 (4.1)
Lung metastasis	
No	209 (71.1)
Yes	85 (28.9)
Subtype of primary site	
HR+/HER2‐	173 (58.8)
HR+/HER2+	29 (9.9)
HR‐/HER2+	29 (9.9)
HR‐/HER2‐	31 (10.5)
Unknown	32 (10.9)
Subtype of liver metastasis site	
HR+/HER2‐	171 (58.2)
HR+/HER2+	28 (9.5)
HR‐/HER2+	31 (10.5)
HR‐/HER2‐	59 (20.1)
Unknown	5 (1.7)

### 3.2. Metastases to Other Organs, HER2+ Subtype and Triple‐Negative Subtype Are Related to a Larger Likelihood of Liver Metastasis

Table 3 summarizes the multifactor logistic regression for BCLM. Younger age, higher pathological grade, bone, lung or brain metastasis, and the HR+/HER2+, HR−/HER2+, and TNBC subtypes were recognized as risk factors for the occurrence of liver metastasis.

**TABLE 3:** Multivariate logistic regression for the risk factors of liver metastases at initial diagnosis of breast cancer in the SEER database.

Variable	Liver metastasis		OR (95% CI)
	No (*N* = 553,661)	Yes (*N* = 7247)	
Age			
18–40	38,981 (7%)	956 (13.2%)	[1]
41–60	234,910 (42.4%)	3236 (44.7%)	**0.70 (0.64–0.76, p < 0.001)**
61–80	239,729 (43.3%)	2575 (35.5%)	**0.52 (0.48–0.57, p < 0.001)**
> 80	40,041 (7.2%)	480 (6.6%)	**0.52 (0.46–0.59, p < 0.001)**
Race			
White	354,915 (64.1%)	4266 (58.9%)	[1]
Black	59,709 (10.8%)	1285 (17.7%)	**1.15 (1.06–1.24, p < 0.001)**
Hispanic	75,781 (13.7%)	976 (13.5%)	**0.85 (0.79–0.93, p < 0.001)**
Asian or Pacific Islander	56,348 (10.2%)	656 (9.1%)	**0.89 (0.81–0.97, p = 0.011)**
American Indian/Alaska Native	3623 (0.7%)	44 (0.6%)	0.75 (0.54–1.06, *p* = 0.104)
Unknown	3285 (0.6%)	20 (0.3%)	0.54 (0.34–0.86, *p* = 0.010)
Marital			
Unmarried	218,979 (39.6%)	3662 (50.5%)	[1]
Married	308,942 (55.8%)	3251 (44.9%)	**0.86 (0.82–0.91, p < 0.001)**
Unknown	25,740 (4.6%)	334 (4.6%)	0.97 (0.85–1.10, *p* = 0.658)
Histology			
Infiltrating duct carcinoma	429,533 (77.6%)	5469 (75.5%)	[1]
Lobular carcinoma	51,430 (9.3%)	445 (6.1%)	1.05 (0.94–1.17, *p* = 0.394)
Infiltrating duct and lobular carcinoma	25,575 (4.6%)	220 (3%)	1.06 (0.91–1.23, *p* = 0.469)
Other types	47,123 (8.5%)	1113 (15.4%)	1.26 (1.16–1.37, *p* < 0.001)
Grade			
I	112,811 (20.4%)	153 (2.1%)	[1]
II	211,832 (38.3%)	1286 (17.7%)	**2.66 (2.24–3.16, p < 0.001)**
III/IV	132,429 (23.9%)	2062 (28.5%)	**4.28 (3.60–5.08, p < 0.001)**
Unknown	96,589 (17.4%)	3746 (51.7%)	5.65 (4.77–6.70, *p* < 0.001)
Bone metastasis			
Yes	15,141 (2.7%)	4461 (61.6%)	1 [Reference]
No	538,520 (97.3%)	2786 (38.4%)	**0.04 (0.04–0.04, p < 0.001)**
Brain metastasis			
Yes	1430 (0.3%)	704 (9.7%)	1 [Reference]
No	552,231 (99.7%)	6543 (90.3%)	**0.44 (0.39–0.50, p < 0.001)**
Lung metastasis			
Yes	6232 (1.1%)	2427 (33.5%)	1 [Reference]
No	547,429 (98.9%)	4820 (66.5%)	**0.20 (0.19–0.22, p < 0.001)**
Subtype			
HR+/HER2−	386,822 (69.9%)	2983 (41.2%)	1 [Reference]
HR+/HER2+	56,521 (10.2%)	1534 (21.2%)	**2.43 (2.25–2.61, p < 0.001)**
HR−/HER2+	23,679 (4.3%)	1099 (15.2%)	**4.60 (4.22–5.01, p < 0.001)**
Triple‐negative	58,809 (10.6%)	1017 (14%)	**1.79 (1.64–1.95, p < 0.001)**
Unknown	27,830 (5%)	614 (8.5%)	1.59 (1.43–1.77, *p* < 0.001)

*Note:* Bold values indicate statistically significant associations in the multivariate logistic regression analysis (*p* < 0.05).

Compared with patients aged 18–40 years, those aged 41–60, 61–80, and > 80 years had significantly lower risks, with ORs of 0.70 (95% CI: 0.64–0.76), 0.52 (0.48–0.57), and 0.52 (0.46–0.59) (all *p* < 0.001), respectively. Compared with white patients, black patients had a higher risk of BCLM (OR = 1.15; 95% CI: 1.06–1.24; *p* < 0.001), whereas Hispanic (0.85; 0.79–0.93; < 0.001) and Asian or Pacific Islander patients (0.89; 0.81–0.97; 0.011) had lower risks. Married patients exhibited a significantly reduced risk of BCLM relative to unmarried patients (OR = 0.86; 95% CI: 0.82–0.91; *p* < 0.001). Patients with grade II (OR = 2.66; 95% CI: 2.24–3.16; *p* < 0.001) and grade III/IV (4.28; 3.60–5.08; < 0.001) tumors versus grade I had markedly higher risks of developing liver metastasis. Patients without metastasis to bone (OR = 0.04; 95% CI: 0.04–0.04; *p* < 0.001), brain (0.44; 0.39–0.50; < 0.001), or lung (0.20; 0.19–0.22; < 0.001) had significantly less risks of BCLM. Patients with HR+/HER2+ (OR = 2.43; 95% CI: 2.25–2.61), HR‐/HER2+ (4.60; 4.22–5.01), and TNBC subtypes (1.79; 1.64–1.95) (all *p* < 0.001) relative to the HR+/HER2‐ subtype had significantly increased risks of liver metastasis.

### 3.3. Metastases to Other Organs and HER2‐ Subtypes Are Associated With High Mortality Risk

**FIGURE 2:**
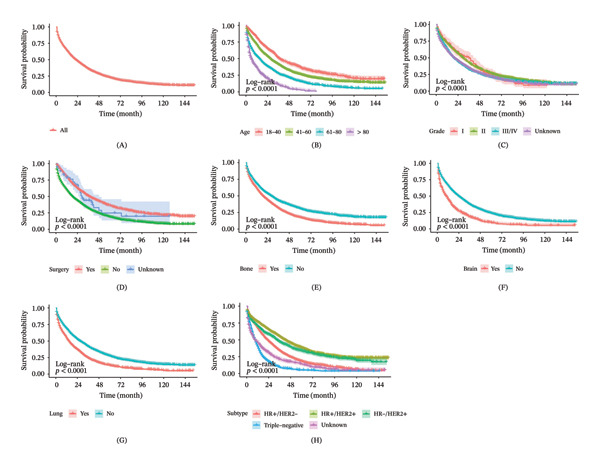
Survival of BCLM patients from the SEER database. (A) OS of all BCLM patients. (B) OS stratified by age. (C) OS stratified by grade. (D) OS stratified by surgical status of the primary breast cancer. (E) OS stratified by bone metastasis status. (F) OS stratified by brain metastasis status. (G) OS stratified by lung metastasis status. (H) OS stratified by molecular subtype.

Figure 2 shows the KM survival curves for OS of patients from the SEER database. The mOS for all patients with liver metastasis was 22 months (95% CI: 21–23) (Figure 2A). The median survival time of BCLM patients gradually decreased with increasing age. The mOS for patients aged 18–40, 41–60, 61–80, and > 80 years was 38 (95% CI: 34–43), 28 (26–29), 16 (14–17), and 7 months (5–8), respectively (Figure 2B). The mOS for patients with histological grades I, II, and III/IV was 36 (95% CI: 25–43), 30 (28–32), and 20 (18–21) months, respectively (Figure 2C). Patients who underwent surgery for the primary tumor had mOS of 37 months (95% CI: 34–41) compared with 19 months (18–20) for those without surgery (Figure 2D). The occurrence of additional organ metastases was linked with significantly shorter survival. Patients with versus without bone metastasis had mOS of 19 (95% CI: 18–20) and 28 (26–31) months, respectively (Figure 2E). Patients with versus without brain metastasis had mOS of 10 (95% CI: 9–12) and 24 (23–25) months, respectively (Figure 2F). Patients with versus without lung metastasis had mOS of 15 (95% CI: 14–16) and 27 (26–29) months, respectively (Figure 2G). Patients with the HER2‐ subtype had shorter survival. The mOS for HR+/HER2‐ and TNBC BCLM patients was 23 (95% CI: 21–24) and 9 (8–10) months, respectively. The mOS for HR+/HER2+ and HR‐/HER2+ subtypes was 42 (95% CI: 39–46) and 34 (31–37) months, respectively (Figure 2H).

**FIGURE 3:**
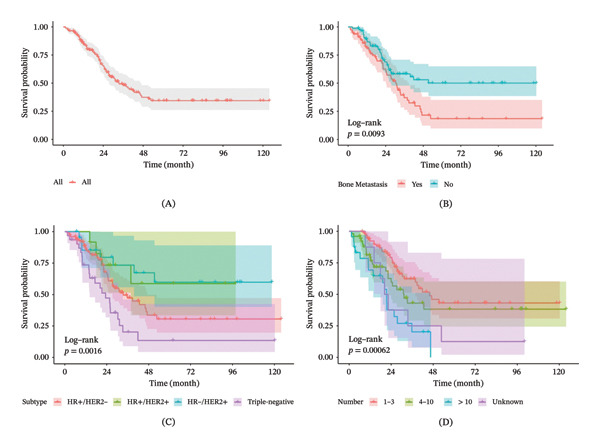
Survival of BCLM patients from the JSPH database. (A) OS of all BCLM patients. (B) OS stratified by bone metastasis status. (C) OS stratified by molecular subtype. (D) OS stratified by number of liver metastatic lesions.

Figure 3 presents the KM survival curves for OS in patients from the JSPH database. The mOS for all BCLM patients was 33.5 months (95% CI: 27.3–46.5) (Figure 3A). Similar to SEER data, patients with metastasis to other organs had largely shorter survival in the JSPH database. The difference in survival was significant between patients with and without bone metastasis, but not for brain and lung metastases, possibly due to small sample sizes. Patients with versus without bone metastasis had mOS of 30.4 months (95% CI: 24–38.8) versus 51 months (Figure 3B). The mOS was not significantly different across primary tumor subtypes, but across the molecular subtypes of liver metastasis, highlighting the prognostic relevance of metastatic lesion subtypes. The mOS of BCLM patients with HR+/HER2‐ and triple negative subtypes was 33.5 (95% CI: 27.3–47.1) and 23.4 (14.2–33.2) months, respectively. The mOS for HR+/HER2+ and HR‐/HER2+ subtypes was not reached (Figure 3C). In addition, the JSPH database analysis revealed that a greater count of liver metastatic sites was associated with poorer prognosis. Patients with 1–3, 4–10, and > 10 lesions had mOS of 46 (95%CI: 37.8–NR), 31.3 (22.3–NR), and 21.4 (13.8–NR) months, respectively (Figure 3D).

We further conducted univariate and multivariate Cox regressions to seek prognostic factors of BCLM. In the SEER database, advanced age, unmarried status, higher pathological grade, lack of primary tumor surgery, presence of metastases in other organs, and HER2‐subtype were related to a higher risk of death (Table 4), which accords with the KM analysis. In the JSPH database, BCLM patients with concomitant bone metastasis, no surgical resection of hepatic metastatic lesions, HER2‐ subtype in liver metastasis, and a higher number of liver metastatic lesions exhibited a higher risk of mortality (Table 5).

**TABLE 4:** Univariate and multivariate cox regression for OS of breast cancer with liver metastases at diagnosis in the SEER database.

Variable	HR (univariable)	HR (multivariable)
Age		
18–40	1 [Reference]	1 [Reference]
41–60	1.36 (1.24–1.50, *p* < 0.001)	**1.27 (1.15-1.39, *p* < 0.001)**
61–80	2.09 (1.90–2.30, *p* < 0.001)	**1.70 (1.54-1.87, *p* < 0.001)**
> 80	3.68 (3.24–4.19, *p* < 0.001)	**2.39 (2.08-2.74, *p* < 0.001)**
Race		
White	1 [Reference]	1 [Reference]
Black	1.30 (1.21–1.40, *p* < 0.001)	1.29 (1.19–1.39, *p* < 0.001)
Hispanic	0.95 (0.87–1.03, *p* = 0.204)	1.02 (0.94–1.12, *p* = 0.600)
Asian or Pacific Islander	0.77 (0.70–0.86, *p* < 0.001)	0.83 (0.75–0.93, *p* = 0.001)
American Indian/Alaska Native	1.27 (0.92–1.77, *p* = 0.149)	1.27 (0.91–1.77, *p* = 0.156)
Unknown	0.55 (0.26–1.16, *p* = 0.116)	0.53 (0.25–1.11, *p* = 0.093)
Marital		
Unmarried	1 [Reference]	1 [Reference]
Married	0.75 (0.71–0.79, *p* < 0.001)	**0.89 (0.84–0.94, *p* < 0.001)**
Unknown	1.00 (0.88–1.13, *p* = 0.959)	0.92 (0.81–1.05, *p* = 0.205)
Histology		
Infiltrating duct carcinoma	1 [Reference]	1 [Reference]
Lobular carcinoma	1.16 (1.04–1.30, *p* = 0.010)	1.07 (0.95–1.21, *p* = 0.235)
Infiltrating duct and lobular carcinoma	0.94 (0.80–1.10, *p* = 0.417)	1.05 (0.89–1.24, *p* = 0.535)
Other types	1.52 (1.41–1.63, *p* < 0.001)	1.19 (1.10–1.29, *p* < 0.001)
Grade		
I	1 [Reference]	1 [Reference]
II	0.97 (0.81–1.17, *p* = 0.786)	**1.26 (1.05–1.52, *p* = 0.015)**
III/IV	1.17 (0.97–1.40, *p* = 0.096)	**1.62 (1.34–1.95, *p* < 0.001)**
Unknown	1.21 (1.01–1.45, *p* = 0.043)	**1.34 (1.11–1.61, *p* = 0.002)**
Surgery of primary site		
Yes	1 [Reference]	1 [Reference]
No	1.74 (1.62–1.87, *p* < 0.001)	**1.44 (1.33–1.55, *p* < 0.001)**
Unknown	1.15 (0.81–1.64, *p* = 0.428)	1.08 (0.75–1.53, *p* = 0.687)
Radiotherapy		
Yes	1 [Reference]	
No/unknown	1.04 (0.98–1.11, *p* = 0.185)	
Chemotherapy		
Yes	1 [Reference]	1 [Reference]
No/unknown	2.30 (2.17–2.44, *p* < 0.001)	1.94 (1.82–2.07, *p* < 0.001)
Bone metastasis		
Yes	1 [Reference]	1 [Reference]
No	0.70 (0.66–0.74, *p* < 0.001)	**0.75 (0.70–0.79, *p* < 0.001)**
Brain metastasis		
Yes	1 [Reference]	1 [Reference]
No	0.56 (0.51–0.61, *p* < 0.001)	**0.63 (0.58–0.69, *p* < 0.001)**
Lung metastasis		
Yes	1 [Reference]	1 [Reference]
No	0.63 (0.59–0.67, *p* < 0.001)	**0.79 (0.74–0.83, *p* < 0.001)**
Subtype		
HR+/HER2‐	1 [Reference]	1 [Reference]
HR+/HER2+	0.59 (0.55–0.64, *p* < 0.001)	**0.70 (0.65–0.76, *p* < 0.001)**
HR‐/HER2+	0.70 (0.64–0.77, *p* < 0.001)	**0.91 (0.83–0.99, *p* = 0.037)**
Triple‐negative	2.00 (1.85–2.17, *p* < 0.001)	**2.47 (2.27–2.69, *p* < 0.001)**
Unknown	1.51 (1.38–1.67, *p* < 0.001)	1.34 (1.21–1.48, *p* < 0.001)

*Note:* Bold values indicate statistically significant results in the multivariate cox regression analysis (*p* < 0.05).

**TABLE 5:** Univariate and multivariate cox regression for OS after the diagnosis of liver metastasis in breast cancer patients in the JSPH database.

Variable	HR (univariable)	HR (multivariable)
Age		
18–40	1 [Reference]	1 [Reference]
41–60	0.82 (0.48–1.38, *p* = 0.449)	1.36 (0.70–2.65, *p* = 0.365)
61–80	0.67 (0.31–1.44, *p* = 0.302)	1.03 (0.41–2.55, *p* = 0.955)
Surgery of primary site		
Yes	1 [Reference]	1 [Reference]
No	0.31 (0.08–1.26, *p* = 0.102)	0.43 (0.10–1.88, *p* = 0.261)
Surgery of liver metastasis site		
Yes	1 [Reference]	1 [Reference]
No	2.07 (1.00–4.31, *p* = 0.051)	**2.44 (1.02–5.84, *p* = 0.046)**
Radiotherapy		
Yes	1 [Reference]	1 [Reference]
No/unknown	0.62 (0.39–0.97, *p* = 0.036)	0.57 (0.33–0.96, *p* = 0.036)
Bone metastasis		
Yes	1 [Reference]	1 [Reference]
No	0.56 (0.36–0.88, *p* = 0.011)	**0.65 (0.37–1.16, *p* = 0.145)**
Brain metastasis		
Yes	1 [Reference]	1 [Reference]
No	1.36 (0.19–9.82, *p* = 0.763)	3.26 (0.43–24.64, *p* = 0.251)
Lung metastasis		
Yes	1 [Reference]	1 [Reference]
No	0.78 (0.48–1.27, *p* = 0.317)	0.90 (0.51–1.61, *p* = 0.730)
Subtype of primary site		
HR+/HER2‐	1 [Reference]	1 [Reference]
HR+/HER2+	0.63 (0.30–1.35, *p* = 0.234)	0.73 (0.28–1.94, *p* = 0.532)
HR‐/HER2+	0.79 (0.38–1.61, *p* = 0.509)	1.14 (0.42–3.14, *p* = 0.796)
Triple‐negative	1.32 (0.70–2.49, *p* = 0.395)	1.54 (0.64–3.73, *p* = 0.335)
Unknown	0.43 (0.15–1.20, *p* = 0.106)	0.39 (0.11–1.32, *p* = 0.129)
Subtype of liver metastasis		
HR+/HER2‐	1 [Reference]	1 [Reference]
HR+/HER2+	0.53 (0.19–1.48, *p* = 0.229)	0.70 (0.22–2.21, *p* = 0.542)
HR‐/HER2+	0.48 (0.21–1.07, *p* = 0.072)	**0.54 (0.18–1.61, *p* = 0.272)**
Triple‐negative	1.95 (1.17–3.24, *p* = 0.010)	**2.01 (0.97–4.18, *p* = 0.061)**
Number of liver metastases		
1–3	1 [Reference]	1 [Reference]
4–10	1.55 (0.91–2.66, *p* = 0.107)	1.36 (0.74–2.51, *p* = 0.322)
> 10	3.15 (1.74–5.71, *p* = 0.001)	**3.09 (1.57–6.09, *p* = 0.001)**
Unknown	2.46 (1.09–5.58, *p* = 0.031)	2.95 (1.12–7.79, *p* = 0.029)

*Note:* Bold values indicate variables of potential prognostic relevance.

### 3.4. Resection of Liver Metastases Improves Survival

**FIGURE 4:**
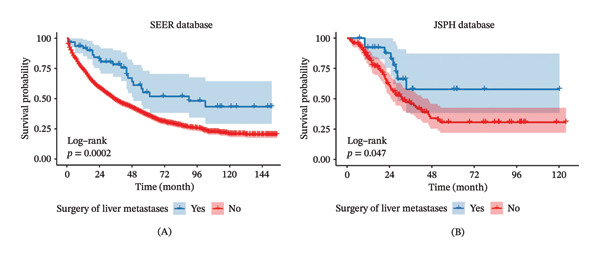
(A) OS stratified by resection of liver metastases from the SEER database. (B) OS stratified by resection of liver metastases from the JSPH database.

In the SEER database, among 7247 BCLM patients, we excluded those with concurrent bone, brain, or lung metastasis, leaving 2038 patients with liver‐only metastasis. These patients were then stratified into a liver metastasectomy group and a nonsurgery group for survival analysis. The surgery group had significantly longer mOS than the nonsurgery group (90 months (95% CI: 27.3–NR) vs. 35 months (32–39)) (Figure 4A). Similar results were observed in the JSPH database, where the mOS was not reached after liver metastasectomy, while the nonsurgery patients had mOS of 31.3 months (95% CI: 25–46) (Figure 4B).

### 3.5. Receptor Conversion

**FIGURE 5:**
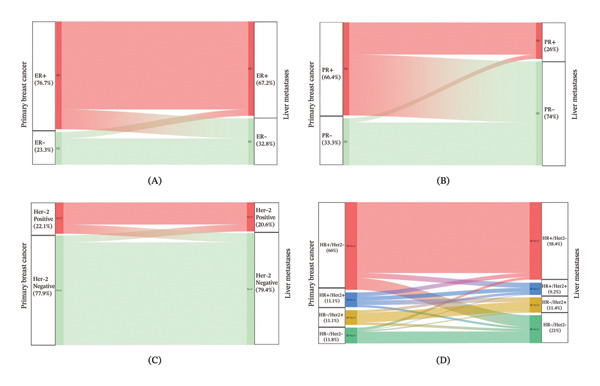
Receptor conversion from primary breast cancer to BCLM. (A) ER conversion. (B) PR conversion. (C) HER2 conversion. (D) Subtype conversion.

In the JSPH database, receptor status can change during the metastatic process, and such receptor conversion is closely associated with prognosis. Among 262 patients with complete molecular subtype records for both primary tumors and metastatic lesions, we analyzed receptor changes. ER expression changed in 49/262 patients (18.7%), from positive to negative ER in 37/49 (75.5%) and from negative to positive ER in 12/49 (24.5%) (Figure 5A). PR expression changed in 122/262 patients (46.6%), from positive to negative PR in 114/122 (93.4%) and from negative to positive PR in 8/122 (6.6%) (Figure 5B). HER2 expression changed in 30/262 patients (11.5%), from positive to negative HER2 in 17/30 (56.7%) and from negative to positive HER2 in 13/30 (43.3%) (Figure 5C). Overall, 72/262 patients (27.5%) experienced a molecular subtype conversion (Figure 5D).

## 4. Discussion

The clinical characteristics, prognostic factors, and molecular subtype dynamics of BCLM were systematically analyzed using the SEER and JSPH databases. Liver metastasis attacks 1.3% of newly diagnosed breast cancer patients, ranking third after bone (3.5%) and lung (1.5%) metastases. Among all molecular subtypes, HR‐/HER2+ patients have the highest risk of liver metastasis, indicating that HER2+ tumors are prone to hepatic involvement.

Multivariate analysis shows young age, high pathological grade, concurrent bone, lung, or brain metastasis, and HER2+ or TNBC subtypes are independent risk factors for liver metastasis. Race and marital status affect the risk, as black patients show higher incidence and married patients have lower risk, implying tumor biology and sociodemographic factors interact in the occurrence of liver metastasis.

Survival analysis demonstrates poor overall prognosis for BCLM patients, and median survival is shortened with age, higher pathological grade, and the occurrence of other organ metastases. The median OS of 22 months observed in our SEER cohort is higher than the previously reported 14.3 months. This difference may be explained by several factors, including different study periods, with our analysis covering more recent years during which systemic therapies—especially HER2‐targeted agents and CDK4/6 inhibitors—have been widely adopted. In addition, variations in cohort composition, inclusion criteria, and the population‐based nature of SEER compared with smaller single‐center cohorts may also contribute to the discrepancy in survival estimates. HER2+ patients showed significantly longer survival compared to HER2− and TNBC patients. However, due to the lack of detailed treatment information in the SEER database, it is not possible to determine whether this survival advantage is attributable to intrinsic tumor biology or the effects of systemic therapies, including HER2‐targeted treatment. JSPH data further show that both the number and molecular subtype of liver metastasis can strongly predict survival, indicating that the biological characteristics of metastatic lesions versus primary tumors more directly reflect tumor aggressiveness and treatment response.

Although hepatic resection was associated with significantly improved survival in both cohorts, this finding should be interpreted with caution. Patients selected for surgery are typically those with better performance status, limited liver tumor burden, and more favorable disease biology, which may introduce substantial selection bias. Due to the retrospective nature of this study and the lack of detailed clinical variables in the SEER database, advanced methods such as propensity score matching were not feasible. In real‐world clinical practice, local‐regional treatment strategies for BCLMs extend beyond surgical resection. Modalities such as radiofrequency ablation, stereotactic body radiotherapy, and transarterial chemoembolization have been increasingly used, especially for patients who are not surgical candidates. These approaches may provide local disease control and potential survival benefits in selected patients. Recent real‐world evidence highlights the importance of integrating systemic therapy with local treatment strategies, particularly in HR+/HER2‐ patients, where liver metastasis status may influence first‐line treatment decisions and overall outcomes [16]. Therefore, the survival benefit observed in our study likely reflects both treatment selection and underlying disease characteristics, rather than the independent effect of surgery alone.

Molecular subtype is converted in about 27.5% of patients during disease progression, mainly from positive to negative ER/PR, which may contribute to endocrine therapy resistance and highlight the importance of receptor status re‐evaluation at recurrence or metastatic stage.

This study has several limitations. The definition of overall survival differs between the SEER and JSPH cohorts. In the SEER database, OS was calculated from the time of initial breast cancer diagnosis, whereas in the JSPH cohort, it was defined from the time of liver metastasis diagnosis. This methodological inconsistency limits the direct comparability of survival outcomes between the two cohorts and may partially contribute to the longer median OS observed in the JSPH cohort. Several limitations should be acknowledged, including the lack of detailed treatment information and HER2‐targeted therapy records in the SEER database, the relatively small sample size of the JSPH cohort, which may have limited the statistical power of certain subgroup analyses, and the retrospective study design, which may have introduced selection bias. Prospective research is needed to verify the independent impacts of liver metastasis resection and metastatic lesion molecular subtypes on prognosis.

## 5. Conclusions

The occurrence of liver metastasis in breast cancer is closely associated with younger age, higher pathological grade, presence of other organ metastases, and molecular subtypes (HER2‐positive and triple‐negative). The prognosis of BCLM patients is generally poor, as median survival is significantly affected by age, pathological grade, other organ involvement, and molecular subtype. The survival of patients with positive HER2 or after hepatic resection is markedly improved, implying the potential benefit of targeted therapy and local intervention in selected patients. The number and molecular subtype of liver metastasis are key prognostic factors and should be considered in clinical decision‐making. Molecular subtype is converted in 27.5% of patients during metastasis, indicating that the receptor status at recurrence or metastatic stages shall be reassessed to optimize individualized treatment strategy.

## Author Contributions

Dandan Yu and Chengjun Zhu collected the data and wrote the manuscript. Dandan Yu and Sujin Yang processed data and prepared figures and tables. Dandan Wang and Xiaoxiang Guan were responsible for guidance and financial support. Sujin Yang and Dandan Wang confirm the authenticity of all the raw data.

## Funding

This work was supported by National Natural Science Foundation Youth Fund Cultivation Program of The First Affiliated Hospital of Nanjing Medical University (PY2022030).

## Disclosure

All authors read and approved the final manuscript.

## Ethics Statement

This study was performed with approval from the Ethics Committee of JSPH (2025‐SR‐918).

## Consent

The authors have nothing to report.

## Conflicts of Interest

The authors declare no conflicts of interest.

## Data Availability

Part of the data originated from the SEER database of the National Cancer Institute (https://seer.cancer.gov/) on SEER∗Stat 9.0.41; the remaining data can be requested from the corresponding author.
